# Statins and Myasthenia Gravis: Clinical Implications and Pathogenesis

**DOI:** 10.3390/ijms27188150

**Published:** 2026-09-13

**Authors:** Xiao Tian, Peixiang Zhang

**Affiliations:** Division of Endocrinology, Diabetes and Nutrition, Department of Medicine, University of Maryland School of Medicine, Baltimore, MD 21201, USA; xtian@som.umaryland.edu

**Keywords:** statins, myasthenia gravis, mevalonate pathway, glycosylation, neuromuscular junction

## Abstract

Statins are widely prescribed lipid-lowering agents and remain central to the prevention of atherosclerotic cardiovascular disease. Although generally well tolerated, they have been associated with neuromuscular adverse events, including new-onset or worsening myasthenia gravis (MG). Current evidence supports a primarily temporal association between statin exposure and MG onset or exacerbation; this signal has emerged largely from case reports, pharmacovigilance analyses, and retrospective observational studies, while more recent population-based observational data strengthen the association. Statin-associated MG should be distinguished from other statin-related neuromuscular syndromes, particularly toxic myopathy and immune-mediated necrotizing myopathy. Beyond cholesterol-lowering, statins may influence neuromuscular junction function through multiple converging mechanisms, including perturbation of the mevalonate–dolichol–glycosylation pathway, isoprenoid-dependent immune dysregulation, altered lipid raft integrity and acetylcholine receptor clustering, and coenzyme Q10-linked mitochondrial vulnerability. In this review, we summarize the clinical evidence linking statins to MG, outline key differential diagnostic considerations, and propose an integrated glycosylation–immune–structural–metabolic framework to guide future mechanistic studies and potential risk-stratified clinical management.

## 1. Introduction

Cardiovascular disease remains a leading cause of morbidity and mortality worldwide, and statins remain central to both primary and secondary cardiovascular prevention [1,2,3]. In the United States alone, approximately 91.2 million adults aged 20 years or older have total cholesterol levels above 200 mg/dL, and nearly 11.3% have levels above 240 mg/dL [1]. Statins are widely regarded as safe and effective lipid-lowering agents and are commonly prescribed for hypercholesterolemia, a major risk factor for atherosclerotic cardiovascular disease. Accordingly, statin use is widespread, with about one-quarter of U.S. adults older than 40 years taking a statin [2]. Statins exert their principal pharmacologic effect by inhibiting 3-hydroxy-3-methylglutaryl coenzyme A (HMG-CoA) reductase, the rate-limiting enzyme in cholesterol biosynthesis, thereby reducing intracellular cholesterol synthesis, lowering hepatic cholesterol content, and enhancing clearance of circulating low-density lipoprotein cholesterol [3]. Despite these well-established cardioprotective effects, increasing attention has focused on adverse effects associated with statin therapy, including statin-associated muscle symptoms (SAMS) [4,5], new-onset diabetes mellitus [6,7], and neurologic manifestations involving both the central and peripheral nervous systems [8,9,10].

Potential neurotoxicity associated with statin therapy has emerged as an area of increasing interest. Neurotoxicity can be broadly categorized according to the affected anatomic region into central nervous system (CNS) and peripheral nervous system (PNS) manifestations [10]. CNS-related effects primarily involve the brain and spinal cord and may include memory impairment, cognitive decline, emotional or behavioral changes, and impaired motor coordination [11]. However, the effects of statins on the CNS remain controversial. Some studies have suggested that statin use may be associated with short-term memory impairment or attention deficits [12], whereas others propose neuroprotective effects mediated through activation of AMP-activated protein kinase and improvement of impaired insulin signaling, with potential relevance to neurodegenerative disorders such as Alzheimer’s disease [13]. These divergent findings may reflect differences in statin lipophilicity, blood–brain barrier permeability, and variation in study design and patient populations.

In contrast, the peripheral nervous system may be more vulnerable to statin-related toxicity because of its more direct exposure to circulating statins and their metabolites [14]. Peripheral neurotoxicity may affect sensory, motor, or autonomic nerves and may manifest as numbness, tingling, pain, sensory loss, or muscle weakness [15]. Experimental evidence further suggests that statins may alter cholesterol trafficking within the nervous system. In hypercholesterolemic animal models, atorvastatin has been reported to promote the mobilization of cholesterol to the spinal cord, a process that may contribute to statin-associated peripheral nervous system toxicity. This effect may be related, at least in part, to organic anion-transporting polypeptides, which regulate tissue uptake and distribution of statins and related lipid metabolites [16]. Emerging evidence has also linked statin use to an increased risk of myasthenia gravis, a chronic neuromuscular junction disorder characterized by variable skeletal muscle weakness [17]. Statin therapy has further been associated with small but statistically significant increases in both exacerbations of preexisting myasthenia gravis and incident disease among individuals without prior myasthenia gravis [18].

Myasthenia gravis is an autoimmune disorder of the neuromuscular junction in which impaired communication between nerves and muscles leads to fluctuating skeletal muscle weakness [19]. Disease onset may be abrupt, and weakness typically worsens with exertion and improves with rest. Because myasthenia gravis affects voluntary muscles, it can involve a broad range of muscle groups, most commonly those controlling eye movements, facial expression, speech, swallowing, and limb movement. Consequently, patients often present with diplopia, ptosis, dysarthria, dysphagia, and difficulty walking [20]. Myasthenia gravis is a relatively uncommon but increasingly recognized autoimmune disorder, with a global prevalence of approximately 173 cases per million and a potentially higher burden in recent United States cohorts, particularly among older adults [21]. Recent studies have also begun to define immunologic features associated with disease heterogeneity, including altered galactosylation of serum immunoglobulin G in myasthenia gravis subgroups with different autoantibodies [22].

Emerging clinical observations and case reports have suggested that statin use may be associated with either new-onset myasthenia gravis or exacerbation of preexisting disease [23]. Some patients developed typical manifestations of myasthenia gravis after initiation of statin therapy, including muscle weakness [24,25,26], fatigability, and ptosis [27]. In certain cases, symptoms partially or completely improved after statin discontinuation [28], suggesting that statins may act as a triggering or exacerbating factor in susceptible individuals [29]. Early evidence linking statins to myasthenia gravis was dominated by case reports, small case series, and pharmacovigilance signals describing new-onset or worsening disease after statin initiation, often with improvement after drug withdrawal and occasional recurrence on rechallenge. More recent population-based observational studies have strengthened this association. However, the available evidence remains largely observational and pharmacovigilance-based, with inherent limitations including underreporting, reporting bias, and incomplete clinical confirmation. Thus, definitive causal inference will require well-controlled clinical studies, complemented by experimental work that directly tests plausible biological mechanisms. Current regulatory and guideline language is similarly cautious, describing statins as agents that may rarely precipitate or worsen MG rather than as proven direct causes [18]. Therefore, this review aims to summarize the clinical evidence linking statin use to myasthenia gravis and to explore the potential pathophysiological mechanisms involved, thereby providing a basis for clinical risk assessment and individualized therapeutic decision-making.

## 2. Clinical Evidence Linking Statins and Myasthenia Gravis

Based on their physicochemical and pharmacokinetic properties, statins are generally classified as lipophilic or hydrophilic agents [30]. Lipophilic statins, including simvastatin, atorvastatin, and lovastatin, diffuse more readily across cell membranes and can enter extrahepatic tissues, including skeletal muscle and the nervous system. By contrast, hydrophilic statins, including pravastatin and rosuvastatin, rely more heavily on transporter-mediated uptake into hepatocytes and therefore may have lower exposure in extrahepatic tissues [30]. These differences may partially influence the risk of neurologic and neuromuscular adverse effects and may provide insight into heterogeneity in toxicity among individual statins.

Growing attention has focused on the potential effects of statins on neuromuscular junction function [31]. Fatigable weakness after statin exposure, followed in some patients by a diagnosis of myasthenia gravis or myasthenia gravis-like disease, suggests that statins may unmask or exacerbate impaired neuromuscular transmission in susceptible individuals [32]. This effect is unlikely to reflect cholesterol-lowering alone and may instead involve convergent effects on membrane organization, immune regulation, and cellular metabolism. The reported clinical phenotype typically emerges within weeks to months after statin initiation and includes ptosis [33,34], diplopia, dysphagia, proximal muscle weakness, and marked fatigability [35,36,37,38,39,40,41,42,43,44,45,46,47,48]. In some cases, acetylcholine receptor or muscle-specific kinase antibodies were detected [39], supporting an autoimmune myasthenia gravis phenotype. Importantly, cases have also been described in patients without a prior history of myasthenia gravis, raising the possibility that statin exposure may contribute to disease onset rather than simply exacerbate pre-existing disease [40].

In addition to new-onset disease, statins have also been reported to worsen preexisting myasthenia gravis [41]. Among patients with an established diagnosis, initiation of statin therapy or escalation of dose has been associated with worsening weakness, increased fatigability, and reduced functional capacity. In rare cases, deterioration has progressed to myasthenic crisis, requiring hospitalization and intensification of immunotherapy [42]. Although such severe events appear to be uncommon, their potential clinical consequences are substantial. Notably, several reports have documented partial or marked improvement after discontinuation of statin therapy, sometimes without modification of the existing immunosuppressive regimen. This pattern of temporal association, symptom reversibility, and, in selected cases, recurrence on rechallenge provides indirect but important support for a clinical link between statin exposure and myasthenia gravis (Table 1).

Taken together, the available literature supports a temporal association between statin therapy and either new-onset or worsening myasthenia gravis in a subset of susceptible individuals. However, the overall quality of evidence remains limited, and current data are insufficient to establish a definitive causal relationship. Larger clinical studies and mechanistic investigations will be required to define the magnitude of risk, identify susceptible subgroups, and distinguish statin-associated myasthenia gravis from other statin-related neuromuscular syndromes.

## 3. Differential Diagnosis from Other Statin-Associated Myopathies

When evaluating a possible association between statin therapy and myasthenia gravis, it is essential to distinguish this condition from other statin-associated muscle symptoms. The most common muscle-related adverse effects of statin therapy, affecting up to 20% of users, include myalgia [46,47], myopathy [48], and, in severe cases, rhabdomyolysis [49]. In addition, a rare but clinically important autoimmune myopathy, immune-mediated necrotizing myopathy, has been reported in association with statin exposure. Immune-mediated necrotizing myopathy is characterized by progressive symmetric proximal muscle weakness and markedly elevated creatine kinase levels [50]. Patients frequently test positive for antibodies against 3-hydroxy-3-methylglutaryl coenzyme A reductase [50,51,52], and proximal weakness is a hallmark clinical feature [53].

In inflammatory myopathies, serum creatine kinase does not always correlate precisely with disease activity. For example, some patients with dermatomyositis may have substantial weakness despite normal creatine kinase levels, possibly reflecting muscle fiber atrophy and perivascular inflammation without prominent membrane disruption. By contrast, immune-mediated necrotizing myopathy is driven predominantly by muscle fiber necrosis and is therefore typically associated with markedly elevated creatine kinase during active disease [53]. Muscle biopsy in immune-mediated necrotizing myopathy usually demonstrates extensive necrotic fibers with macrophage infiltration and evidence of regeneration [54]. Unlike common statin-associated myalgia or nonimmune toxic myopathy, symptoms of immune-mediated necrotizing myopathy may persist or even progress after statin withdrawal and often require immunosuppressive treatment [50]. Although rare, immune-mediated necrotizing myopathy carries substantial clinical severity (Table 2).

By contrast, myasthenia gravis is an autoimmune disorder of the neuromuscular junction characterized by impaired neuromuscular transmission rather than structural destruction of muscle fibers. The primary pathogenic mechanism involves functional impairment or reduced numbers of acetylcholine receptors at the postsynaptic membrane. Clinically, myasthenia gravis typically presents with ptosis, diplopia, and fatigable weakness that worsens with activity and improves with rest. Patients often test positive for acetylcholine receptor or muscle-specific kinase antibodies [55]. In laboratory evaluation, creatine kinase levels are usually normal or only mildly elevated, which helps distinguish myasthenia gravis from primary myopathies [56]. Accordingly, when statin-treated patients present with weakness, clinicians should distinguish primary myopathy from neuromuscular junction dysfunction by considering the pattern of symptoms, particularly fatigability and fluctuation, together with creatine kinase levels, autoantibody testing, and electrophysiologic findings. Accurate differentiation is critical for avoiding misdiagnosis and for guiding decisions regarding statin discontinuation, further diagnostic testing, and the initiation of immunotherapy.

## 4. Potential Mechanisms of Statin-Associated Myasthenia Gravis

Genetic background is an important modifier of statin-associated neuromuscular toxicity. The best-established pharmacogenetic example is *SLCO1B1*, which encodes the hepatic organic anion transporting polypeptide OATP1B1 and influences hepatic statin uptake, systemic exposure, and risk of statin-induced myopathy [4,5]. Genome-wide association and pharmacogenomic studies have shown that *SLCO1B1* variants, particularly reduced-function alleles, increase susceptibility to statin-associated muscle injury [4,5]. However, the relevance of *SLCO1B1* to statin-associated myasthenia gravis remains unclear, as current evidence primarily links this locus to statin myopathy rather than autoimmune neuromuscular junction disease. The genetic determinants of susceptibility to statin-associated myasthenia gravis remain poorly defined. Below, we review potential mechanisms by which statins may interact with neuromuscular junction vulnerability and immune regulation.

Statins lower cholesterol primarily through competitive inhibition of 3-hydroxy-3-methylglutaryl coenzyme A reductase, the rate-limiting enzyme of the mevalonate pathway [57]. The resulting decline in intracellular cholesterol induces low-density lipoprotein receptor upregulation in hepatocytes and enhances clearance of circulating low-density lipoprotein cholesterol, which underlies the lipid-lowering and cardiovascular protective effects of these agents [3]. However, the mevalonate pathway also generates nonsterol isoprenoid products, including farnesyl pyrophosphate, geranylgeranyl pyrophosphate, dolichol, and ubiquinone [58,59]. Depletion of these intermediates may affect cellular homeostasis through mechanisms extending beyond cholesterol-lowering.

Mevalonate-derived farnesyl pyrophosphate and geranylgeranyl pyrophosphate are required for prenylation of Ras-superfamily small GTP-binding proteins, enabling correct membrane localization and signaling functions that regulate immune signaling, membrane organization, and cytoskeletal dynamics [60,61]. In parallel, dolichol serves as a critical lipid carrier for protein glycosylation, whereas ubiquinone supports mitochondrial electron transport [62,63,64]. Dolichol is generated by sequential elongation of farnesyl diphosphate with isopentenyl diphosphate units by cis-prenyltransferases, including human dehydrodolichyl diphosphate synthase, followed by dephosphorylation and saturation of the terminal α-isoprenoid unit [65]. By contrast, prenyl diphosphate synthase 1 and prenyl diphosphate synthase 2 are branch-point enzymes required for elongation of the ubiquinone side chain and coenzyme Q biosynthesis [66]. Thus, statin-mediated perturbation of these nonsterol branches may converge on immune dysregulation, neuromuscular junction instability, and impaired cellular energetics in susceptible individuals.

### 4.1. Statin-Induced Perturbation of the Mevalonate–Dolichol Pathway May Intersect with Glycosylation Defects

Current evidence supports a subtype-specific pathogenic framework in myasthenia gravis, shaped by both inherited susceptibility and acquired environmental influences, in which autoantibodies impair neuromuscular transmission through distinct mechanisms. In acetylcholine receptor-positive myasthenia gravis, pathogenic immunoglobulin G, predominantly immunoglobulin G1 and immunoglobulin G3, promotes acetylcholine receptor cross-linking and internalization and induces classical complement-mediated injury at the postsynaptic membrane. In muscle-specific kinase-positive myasthenia gravis, pathogenic immunoglobulin G4 disrupts the agrin–low-density lipoprotein receptor-related protein 4-muscle-specific kinase signaling axis required for acetylcholine receptor clustering and typically does not depend on classical complement activation. Importantly, immunoglobulin G effector function is also conditioned by glycosylation. Fc glycan composition can modulate Fcγ receptor engagement and complement activation, with Fc galactosylation promoting C1q binding and Fc sialylation attenuating complement-dependent effector activity. In parallel, variable-region N-glycosylation motifs are enriched in myasthenia gravis B-cell repertoires, suggesting that glycosylation may also influence autoreactive clone selection and persistence even when it is not strictly required for autoantigen binding. Together, these observations position glycosylation as a potentially important control point in antibody-mediated pathogenicity in myasthenia gravis.

**Figure 1:**
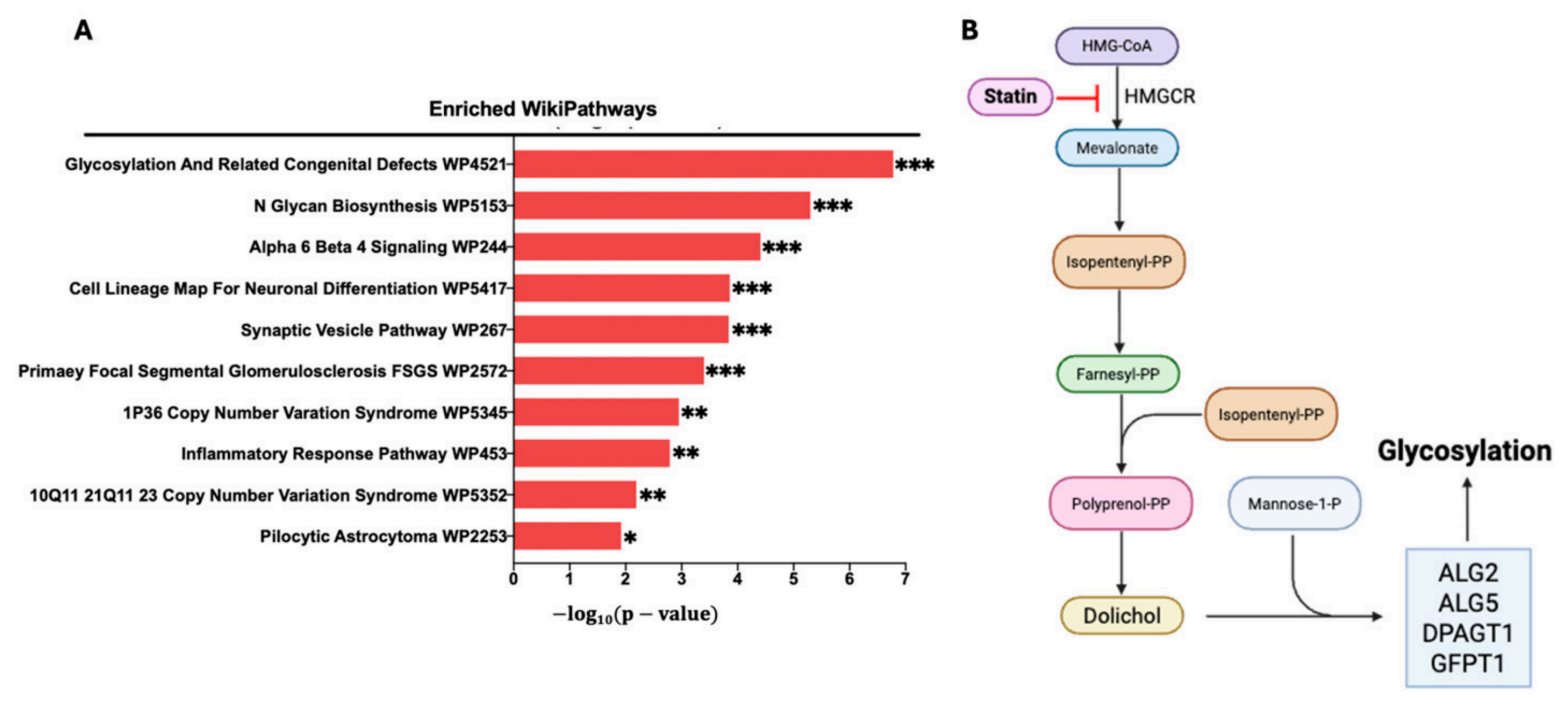
Pathway enrichment analysis and schematic overview of the statin-linked dolichol–glycosylation pathway: (**A**) Pathway enrichment analysis of genes implicated in congenital myasthenic syndromes. Bars are ranked by enrichment score (−log_10_ (*p*-value)). The x-axis indicates the enrichment score, and the y-axis lists the significantly enriched pathways. Asterisks indicate the significance level of pathway enrichment. (**B**) Schematic overview of the mevalonate–dolichol glycosylation axis. Statins inhibit HMG-CoA reductase, thereby reducing flux from HMG-CoA to mevalonate and downstream intermediates, including isopentenyl pyrophosphate, farnesyl pyrophosphate, polyprenyl pyrophosphate, and dolichol. The diagram also highlights the involvement of mannose-1-phosphate and glycosylation-related genes, including *ALG2*, *ALG5*, *DPAGT1*, and *GFPT1*, in glycan assembly. *, *p* < 0.05; **, *p* < 0.01; ***, *p* < 0.001.

A complementary line of evidence comes from congenital myasthenic syndromes, which arise from germline defects in proteins required for neuromuscular junction development, maintenance, or transmission. To date, 40 genes have been implicated, including *AGRN*, *ALG14*, *ALG2*, *CHAT*, *CHD8*, *CHRNA1*, *CHRNB1*, *CHRND*, *CHRNE*, *CHRNG*, *COL13A1*, *COLQ*, *DES*, *DOK7*, *DPAGT1*, *GFPT1*, *GMPPB*, *LAMA5*, *LAMB2*, *LRP4*, *MACF1*, *MUSK*, *MYO9A*, *PLEC*, *PREPL*, *PTPN11*, *PURA*, *RAPSN*, *RPH3A*, *SCN4A*, *SLC18A3*, *SLC25A1*, *SLC5A7*, *SNAP25*, *SYT2*, *TEFM*, *TOR1AIP1*, *UNC13A*, *UNC50*, and *VAMP1*. We performed pathway enrichment analysis using Enrichr on these 40 genes and identified N-glycan biosynthesis as the most significantly enriched pathway, with *ALG2*, *ALG14*, *DPAGT1*, *GFPT1*, and *GMPPB* mapping directly to glycosylation-related processes (Figure 1A). These findings indicate that defective glycosylation is a recurring mechanistic theme in inherited disorders of neuromuscular transmission. They also raise the possibility that partial insufficiency or limited reserve within glycosylation pathways may predispose certain individuals to heightened vulnerability when exposed to environmental stressors such as statins, thereby increasing the likelihood of disease onset or exacerbation.

This framework is strengthened by the role of dolichol in protein glycosylation. In addition to cholesterol, the mevalonate pathway generates dolichol, a lipid carrier that is essential for multiple glycosylation reactions. In its diphosphate form, dolichol provides the membrane scaffold on which the lipid-linked oligosaccharide is assembled before transfer en bloc to nascent proteins during N-glycosylation. In its phosphorylated form, dolichol also serves as a carrier for monosaccharides required for N-glycosylation, O-mannosylation, C-mannosylation, and glycosylphosphatidylinositol-anchor biosynthesis. Because dolichol phosphate sugars are required at multiple steps of glycan assembly, the availability of dolichol phosphate is a critical determinant of efficient N-glycosylation [67,68].

Within this context, statin-mediated inhibition of the mevalonate pathway may have consequences that extend beyond cholesterol-lowering. By reducing mevalonate flux, statins could limit the availability of dolichol-linked intermediates required for efficient glycosylation, particularly under conditions of strong pathway suppression or in biologically susceptible hosts. Such a mechanism is especially compelling in light of two converging observations: first, multiple congenital myasthenic syndrome genes participate directly in glycosylation pathways, and second, altered immunoglobulin G glycosylation has been reported in myasthenia gravis and may correlate with disease subtype, severity, or treatment response. Thus, perturbation of the mevalonate–dolichol–glycosylation axis could plausibly influence myasthenia gravis at more than one level: by modifying glycosylation of immune receptors and autoreactive B-cell pathways, by altering Fc glycoforms that shape complement-driving activity of acetylcholine receptor antibodies, and by further destabilizing neuromuscular junction proteins whose integrity depends on proper glycoprotein processing. Collectively, these data support the hypothesis that statin-induced disruption of dolichol-dependent glycosylation may represent one mechanism by which statins intersect with neuromuscular junction vulnerability and contribute to the onset or worsening of myasthenia gravis in susceptible individuals.

### 4.2. Statins May Reshape Immune Homeostasis Through Isoprenoid-Dependent Immune Signaling

In addition to their effects on cholesterol synthesis, statins suppress the production of the nonsterol isoprenoids farnesyl pyrophosphate and geranylgeranyl pyrophosphate, which are required for prenylation of small GTPases that control immune-cell signaling, trafficking, and activation. Because Ras-, Rho-, Rab-, and Rac-family proteins depend on farnesylation or geranylgeranylation for membrane localization and function, statin-mediated depletion of these intermediates can alter immune responses through mechanisms that are not explained by cholesterol-lowering alone [69,70,71]. Consistent with the classical immunomodulatory view of statins, several studies indicate that limiting mevalonate-derived isoprenoids can dampen antigen presentation and T-cell priming. Human myeloid dendritic cells exposed to statins show impaired maturation and reduced T-cell stimulatory capacity, and these effects are reversible with mevalonate or geranylgeranyl pyrophosphate, supporting a specific role for isoprenoid depletion rather than sterol loss alone. Likewise, simvastatin has been shown to suppress MHC class II antigen processing and presentation by impairing endocytic uptake through defective prenylation of Rho- and Rab-family GTPases, again without requiring measurable disruption of lipid rafts. These data support the idea that reduced farnesyl pyrophosphate and geranylgeranyl pyrophosphate availability can weaken antigen-presenting cell function and thereby attenuate adaptive immune activation in many settings [72].

However, an in-depth review of the literature also indicates that loss of isoprenoid availability is not uniformly anti-inflammatory. Inherited or experimental disruption of the mevalonate pathway, particularly depletion of geranylgeranyl pyrophosphate, has been linked to inflammasome activation and increased IL-1β production, providing a model for how mevalonate insufficiency can drive inflammatory dysregulation rather than simple immune suppression [73]. More recent work likewise shows that protein prenylation restrains innate immune activation, such that reduced geranylgeranylation can release inflammatory signaling pathways in a context-dependent manner [73,74]. Together, these observations argue that inhibition of farnesyl pyrophosphate and geranylgeranyl pyrophosphate synthesis may shift inflammatory thresholds bidirectionally, depending on cell type, differentiation state, and baseline immune context.

This context dependence is also evident across myeloid compartments. In freshly isolated monocytes, statins may have little effect on lipopolysaccharide-induced cytokine production, whereas in macrophages their effects can differ substantially and depend on geranylgeranylation-dependent Rac1 signaling. Thus, statin-mediated interference with prenylation does not produce a single uniform inflammatory outcome; rather, it rewires cell-state-specific signaling programs that can either suppress or preserve cytokine output. This distinction is important for autoimmune disease models because a biologically susceptible host may not respond to mevalonate pathway inhibition in the same way as a healthy or purely hyperinflammatory system [75].

Adaptive immune homeostasis may be especially sensitive to this pathway. Regulatory T-cell stability depends in part on intact mevalonate-pathway activity, and mevalonate or geranylgeranyl pyrophosphate can restore function and survival in mevalonate-compromised Treg systems [76]. Similarly, regulatory B-cell IL-10 production requires geranylgeranyl pyrophosphate-dependent signaling through PI3Kδ-AKT-GSK3, and atorvastatin-induced suppression of this pathway can be rescued by exogenous geranylgeranyl pyrophosphate. These findings suggest that reduced geranylgeranyl pyrophosphate availability may weaken immune tolerance by impairing regulatory lymphocyte programs even while suppressing some pro-inflammatory pathways elsewhere [76,77].

In the context of myasthenia gravis, this framework suggests that statins may not simply suppress immunity but may instead recalibrate immune balance in a manner that becomes maladaptive in susceptible individuals [78]. By perturbing prenylation-dependent signaling in antigen-presenting cells, T cells, Tregs, and regulatory B cells, reduced farnesyl pyrophosphate and geranylgeranyl pyrophosphate availability could alter the threshold for autoreactive T-cell help, compromise immune tolerance, and indirectly favor pathogenic autoantibody responses [69,79,80,81]. This mechanism is conceptually distinct from the glycosylation-centered pathway discussed above: rather than focusing on dolichol-dependent processing of immune and synaptic glycoproteins, it emphasizes how statin-sensitive isoprenoid depletion can reshape immune-cell signaling architecture and inflammatory set points. Direct evidence in myasthenia gravis remains limited, but this prenylation-based model provides a plausible explanation for why statins may be anti-inflammatory in many individuals yet coincide with disease onset or exacerbation in a susceptible subgroup [23].

### 4.3. Cholesterol Homeostasis May Support Structural Resilience of the Neuromuscular Junction

The neuromuscular junction is a highly specialized synapse whose function depends on precise membrane organization within the postsynaptic apparatus. This membrane is enriched in cholesterol and sphingolipids, which form lipid raft microdomains that act not only as structural scaffolds but also as signaling platforms for receptor localization, cytoskeletal anchoring, and transmembrane signal amplification [82,83]. Membrane cholesterol is therefore critical for preserving postsynaptic membrane integrity, maintaining junctional fold architecture, and stabilizing ion channel and receptor organization required for efficient neuromuscular transmission [84].

A central feature of postsynaptic specialization is the dense clustering of acetylcholine receptors, which depends on coordinated signaling through the agrin–low-density lipoprotein receptor-related protein 4-muscle-specific kinase–rapsyn axis [85]. Agrin activates muscle-specific kinase, which recruits rapsyn and promotes acetylcholine receptor clustering and anchorage to the postsynaptic cytoskeleton [85]. Importantly, the membrane localization and signaling efficiency of this machinery are closely linked to lipid raft integrity [86]. Experimental cholesterol depletion disrupts these microdomains, leading to impaired acetylcholine receptor clustering, abnormal receptor distribution, and degeneration of postsynaptic folds, ultimately lowering the safety factor of neuromuscular transmission [84].

This structural model is relevant to statin-associated myasthenia gravis because statins reduce mevalonate pathway flux and, at least in principle, may influence cholesterol availability in extrahepatic tissues and membrane microdomain composition. Although the degree to which therapeutic statin exposure directly lowers cholesterol content at the neuromuscular junction remains uncertain, even modest alterations in local membrane organization could have functional consequences in a synapse that depends on highly ordered receptor packing and signaling geometry [87]. This possibility may be particularly important in the setting of ongoing autoimmune stress. In acetylcholine receptor antibody-positive myasthenia gravis, antibodies promote receptor cross-linking, internalization, and complement-mediated postsynaptic injury. Under these conditions, any reduction in structural resilience caused by altered membrane microdomains could amplify receptor loss and worsen transmission failure [84].

In parallel, reduced availability of mevalonate-derived isoprenoids may impair prenylation of Rho family GTPases and related regulators of actin dynamics, membrane trafficking, and cytoskeletal remodeling [88]. This adds a second layer of vulnerability beyond cholesterol depletion alone. Thus, statin exposure could theoretically destabilize the neuromuscular junction through combined effects on membrane lipid architecture and cytoskeleton-dependent receptor organization [88]. These mechanisms may not be sufficient to cause disease independently, but they may lower the threshold at which immune-mediated injury becomes clinically manifest.

This structural vulnerability model also complements the glycosylation framework. Proper folding, trafficking, and surface stability of neuromuscular junction proteins depend on intact biosynthetic and posttranslational processing pathways. If statin exposure simultaneously compromises dolichol-dependent glycosylation and perturbs cholesterol-rich membrane domains, the neuromuscular junction may become less able to maintain receptor density and signaling fidelity under immune challenge. In this view, statins do not simply lower cholesterol; rather, they may reduce the structural reserve of the postsynaptic membrane in susceptible individuals, thereby contributing to symptom onset or exacerbation.

### 4.4. Mitochondrial Dysfunction May Reduce Metabolic Reserve at the Neuromuscular Junction

**Figure 2:**
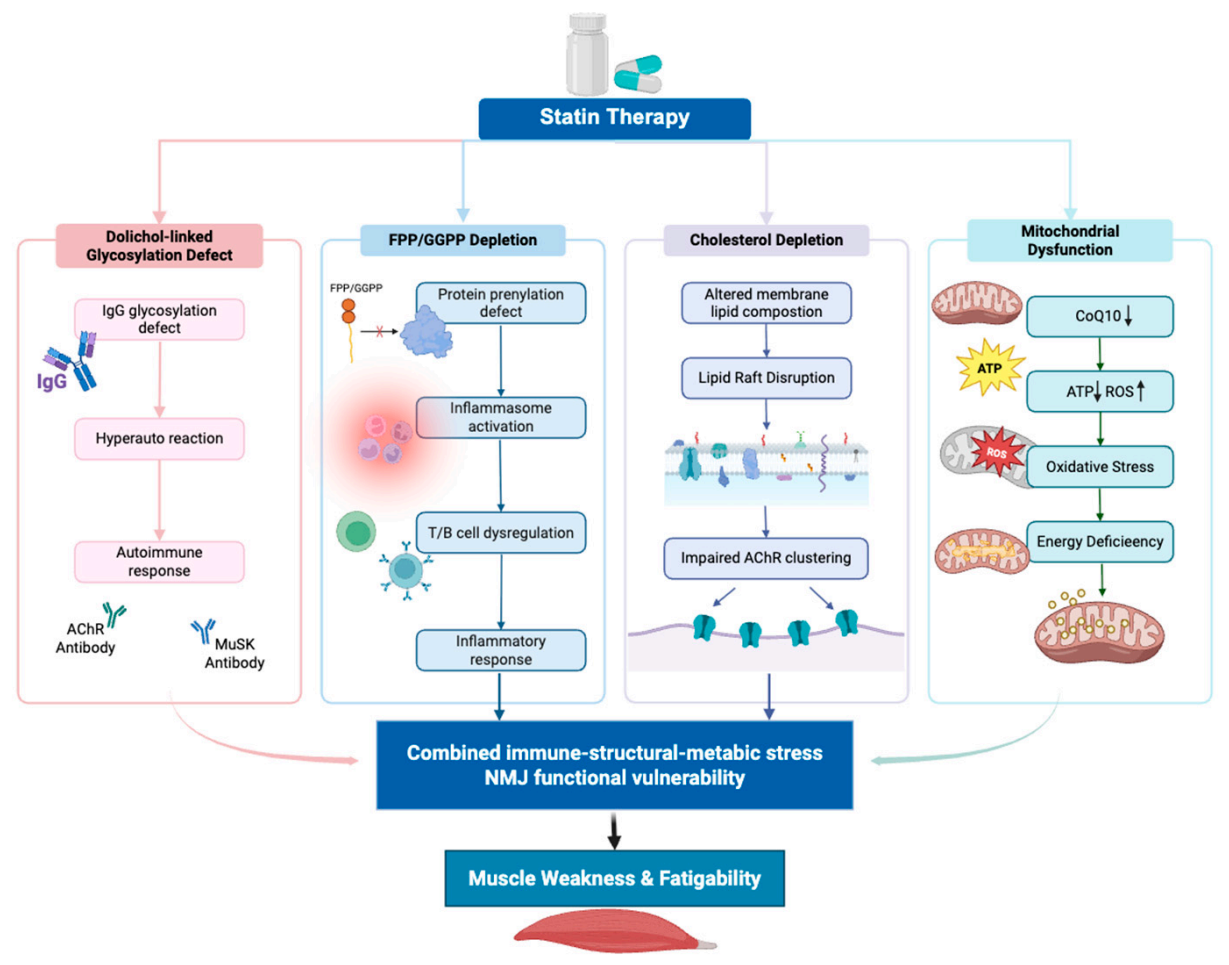
Proposed mechanisms underlying statin-associated myasthenia gravis. Statins inhibit HMG-CoA reductase and reduce flux through the mevalonate pathway, thereby affecting not only cholesterol synthesis but also the production of nonsterol intermediates, including dolichol, farnesyl pyrophosphate, geranylgeranyl pyrophosphate, and coenzyme Q10. In susceptible individuals, these changes may converge on the neuromuscular junction through four interrelated mechanisms. **First**, statin-mediated reduction in mevalonate flux may impair dolichol-dependent glycosylation, thereby altering immune receptor and immunoglobulin G glycoform biology while destabilizing glycoprotein-dependent neuromuscular junction integrity. **Second**, statin-mediated depletion of farnesyl pyrophosphate (FPP) and geranylgeranyl pyrophosphate (GGPP) may alter prenylation-dependent signaling in antigen-presenting cells and regulatory lymphocytes, thereby shifting inflammatory set points, weakening immune tolerance, and facilitating autoreactive responses in susceptible individuals. **Third**, altered cholesterol homeostasis and isoprenoid depletion may disrupt lipid raft integrity, agrin-LRP4-MuSK signaling, acetylcholine receptor clustering, and cytoskeletal organization at the postsynaptic membrane. **Fourth**, reduced coenzyme Q10 may impair mitochondrial respiration, lower ATP production, and increase oxidative stress, thereby diminishing metabolic reserve at both presynaptic and postsynaptic compartments. Together, these structural, immune, and metabolic perturbations may reduce the safety factor of neuromuscular transmission and contribute to the onset or exacerbation of myasthenia gravis. This model is hypothesis-driven and is supported primarily by indirect mechanistic evidence together with clinical observations showing a temporal association between statin exposure and myasthenia gravis in a subset of patients.

A third mechanism by which statins may intersect with myasthenia gravis involves mitochondrial energetics (Figure 2). Coenzyme Q10 is an essential electron carrier in the mitochondrial electron transport chain, transferring electrons between complexes I and III and supporting oxidative phosphorylation and ATP generation [89,90]. Because the isoprenoid side chain of coenzyme Q10 is derived from the mevalonate pathway, inhibition of HMG-CoA reductase may reduce not only cholesterol synthesis but also coenzyme Q10 biosynthesis [91]. Several clinical and experimental studies have reported reductions in circulating or tissue coenzyme Q10 levels during statin therapy, and this has long been considered one potential contributor to statin-associated muscle toxicity [92,93,94].

Mitochondrial effects may also vary by statin type, dose, and experimental context. For example, high-dose rosuvastatin has been reported to alter lipid parameters in hypercholesterolemic mice and to induce marked changes in hepatocyte mitochondrial morphology and function, including respiratory chain impairment and ATP depletion [95]. Although these findings were observed in the liver rather than neuromuscular tissue, they support the broader concept that intensive statin exposure can affect mitochondrial structure and bioenergetic capacity. Such dose-dependent mitochondrial stress may be particularly relevant in individuals with limited neuromuscular reserve.

Reduced coenzyme Q10 availability may impair electron transport efficiency, diminish ATP production, and increase electron leakage with subsequent generation of reactive oxygen species [96]. These changes can promote oxidative stress, disrupt mitochondrial membrane potential, damage mitochondrial DNA, and alter mitochondrial dynamics [97,98]. Such defects are particularly relevant in tissues with high energetic demand, including skeletal muscle and the neuromuscular junction. Maintenance of neuromuscular transmission requires sustained ATP supply for synaptic vesicle loading, vesicle recycling, calcium handling, acetylcholine release, ion gradient maintenance, and postsynaptic signal integration [99]. The neuromuscular junction therefore depends not only on structural integrity but also on adequate metabolic reserve.

At the presynaptic terminal, mitochondrial dysfunction could reduce acetylcholine release by compromising vesicle cycling and calcium homeostasis [99]. At the postsynaptic membrane, diminished ATP availability may impair maintenance of ion gradients and membrane excitability, further lowering the transmission safety factor. In the setting of myasthenia gravis, where receptor loss and complement-mediated membrane injury have already reduced neuromuscular reserve, superimposed energetic insufficiency could have disproportionate functional consequences. This model may help explain why some patients experience fatigability or worsening weakness without extensive structural muscle injury.

Oxidative stress may also intersect with autoimmunity. Reactive oxygen species can drive lipid peroxidation and protein modification, potentially altering antigen structure, increasing epitope exposure, or enhancing local inflammatory signaling [100,101]. In addition, oxidative stress can activate pathways such as NF-κB, thereby amplifying inflammatory responses within vulnerable tissues [102,103]. Although the direct role of these processes in statin-associated myasthenia gravis remains to be established, they provide a plausible link between metabolic stress and immune amplification. Thus, mitochondrial dysfunction may not only reduce the energetic reserve of the neuromuscular junction but may also reinforce local inflammatory and immune-mediated injury.

Taken together, the coenzyme Q10 hypothesis is best viewed as part of a broader convergence model. In susceptible individuals, statin exposure may simultaneously perturb glycosylation, immune homeostasis, membrane organization, and mitochondrial energetics. Each perturbation alone may be modest, but together they could reduce the capacity of the neuromuscular junction to withstand autoimmune stress, thereby precipitating new-onset symptoms or worsening established myasthenia gravis.

## 5. Clinical Implications and Risk-Stratified Considerations

Given the substantial cardiovascular benefit of statins, potential neuromuscular risk should be managed through individualized risk stratification rather than routine discontinuation. This is particularly important in patients at high or very high cardiovascular risk, in whom abrupt withdrawal may increase the likelihood of adverse cardiovascular events. When myasthenia gravis is suspected or already established, treatment decisions should ideally be made through multidisciplinary collaboration between neurologists and cardiovascular specialists. Diagnostic evaluation should first exclude more common statin-associated myopathies and metabolic causes of weakness, supported when appropriate by creatine kinase testing, autoantibody assessment, and electrophysiologic studies.

Closer surveillance may be warranted in patients with lower biologic reserve at the neuromuscular junction or heightened susceptibility to immune dysregulation. These include patients with preexisting myasthenia gravis, especially those with unstable disease activity; individuals with high acetylcholine receptor antibody titers or prior treatment-refractory disease; patients with a history of immune-mediated statin toxicity, including immune-mediated necrotizing myopathy; and, potentially, individuals with inherited or acquired defects in glycosylation pathways that may amplify vulnerability to perturbation of the mevalonate–dolichol–glycosylation axis. In such settings, cautious dose titration, preference for agents with lower extrahepatic tissue exposure, and closer follow-up after treatment initiation or intensification may help reduce risk. For patients who develop recurrent symptom worsening, cannot tolerate rechallenge, or require additional lipid-lowering despite neuromuscular concern, nonstatin therapies such as cholesterol absorption inhibitors or PCSK9-directed agents may be considered.

## 6. Conclusions

Accumulating evidence supports an association between statin exposure and the onset or worsening of myasthenia gravis in a subset of susceptible individuals. One plausible mechanistic model, supported by the enrichment of glycosylation pathways in congenital myasthenic syndromes, is that statin-associated myasthenia gravis arises from preexisting germline susceptibility, particularly variation in glycosylation-related genes, that is unmasked or exacerbated by statin-mediated inhibition of the mevalonate pathway. In this framework, reduced dolichol availability may impair glycoprotein processing, while depletion of isoprenoids, cholesterol, and coenzyme Q10 may further disrupt immune regulation, membrane organization, and mitochondrial energetics.

Together, these perturbations support a multilayered glycosylation–immune–structural–metabolic model in which statins lower the threshold for neuromuscular junction failure in biologically susceptible individuals. Future studies should prioritize the identification of genetic susceptibility factors, glycosylation-related vulnerabilities, and immunologic biomarkers that may improve risk stratification, together with prospective clinical studies to define the magnitude and clinical significance of this association. Overall, the goal should not be routine avoidance of statins, but more precise identification of patients at risk and development of personalized lipid-lowering strategies that preserve cardiovascular benefit while minimizing neuromuscular harm.

## Figures and Tables

**Table 1:** Summary of clinical evidence linking statin therapy and myasthenia gravis.

Statin Type	MG Type	Time to Onset/ Exacerbation	Response After Discontinuation	Rechallenge
Simvastatin	New onset AChR-positive	Several months after initiation	Marked improvement	Yes (symptom recurrence) [43,44]
Rosuvastatin	New onset AChR-positive	Several weeks after initiation	Marked improvement	Not reported [43]
Atorvastatin	Pre-existing	Several weeks after initiation	Partial improvement	No [43,45]
Pravastatin	MG-like symptoms	Several months after initiation	Complete resolution	Yes (symptom recurrence) [43]
Multiple statins	New onset or exacerbated	Several weeks to 1 year	Improvement in most cases	Positive in a few cases [18,39]

Abbreviations: MG, myasthenia gravis; AChR, acetylcholine receptor.

**Table 2:** Key differences between immune-mediated necrotizing myopathy and statin-associated myasthenia gravis.

	IMNM	Statin-Associated MG
Pathogenic mechanism	Direct muscle fiber injury	Impaired neuromuscular junction transmission; autoimmune-mediated
CK level	Markedly elevated	Usually normal or mildly elevated
Antibody testing	Anti-HMGCR antibodies	AChR or MuSK antibodies
Electromyography	Myopathic changes	Neuromuscular transmission defect (typical decrement on repetitive nerve stimulation)
Pattern of muscle weakness	Persistent proximal muscle weakness	Fatigable weakness that worsens after exertion
Response to rest/drug withdrawal	IMNM often requires immunotherapy	May improve after statin withdrawal; primary MG typically requires long-term immunotherapy
Muscle biopsy	Muscle fiber necrosis with minimal inflammation	Changes related to neuromuscular junction pathology

Abbreviations: CK, creatine kinase; IMNM, immune-mediated necrotizing myopathy; anti-HMGCR, against 3-hydroxy-3-methylglutaryl coenzyme A reductase; MuSK, muscle-specific kinase.

## Data Availability

No new data were created or analyzed in this study. Data sharing is not applicable to this article.
